# The complete chloroplast genome sequence of *Gillenia trifoliata* (Rosaceae)

**DOI:** 10.1080/23802359.2019.1639559

**Published:** 2019-07-18

**Authors:** Jiahui Sun, Yiheng Wang, Shiliang Zhou, Luqi Huang

**Affiliations:** a National Resource Center for Chinese Meteria Medica, China Academy of Chinese Medical Sciences, Beijing, China;; b State Key Laboratory of Systematic and Evolutionary Botany, Institute of Botany, Chinese Academy of Sciences, Beijing, China;; c College of Life Sciences, University of Chinese Academy of Sciences, Beijing, China

**Keywords:** *Gillenia* Moench, Maleae, Rosaceae, chloroplast genome, phylogeny

## Abstract

*Gillenia trifoliata* (L.) Moench is an ornamental and medicinal species endemic to North America. In this study, a complete circular chloroplast genome of *G. trifoliata* was assembled and annotated. The chloroplast genome of *G. trifoliata* is 159,496 bp in size and consists of two IR (26,394 bp) regions separated by LSC (87,647 bp) and SSC (19,061 bp) regions. The GC content of the chloroplast genome is 36.5%. The chloroplast genome comprised of 112 different genes, including 78 protein-coding genes. The phylogenetic analysis revealed that *G. trifoliata* lies within subfamily Amygdaloideae and forms the progenitor lineage of the pome-bearing Maleae.

*Gillenia* Moench is a small genus of two species in the family Rosaceae. It was considered to be included in Spiraeoideae traditionally by morphology. However, according to the three-subfamily division system proposed from molecular phylogeny, *Gillenia* apparently comes from the lineage which has closer genetic relationships with tribe Maleae (included in subfamily Amygdaloideae) (Zhang et al. 2017; Sun et al. 2018). *Gillenia trifoliata* (L.) Moench is the type species of *Gillenia*. Common names for *G. trifoliata* include Bowman's root, Indian-physic, and American ipecac. It is a rhizomatous perennial herb with semi-woody branches and 3-palmate leaves and blooms in midsummer with star-shaped white or pale pink flowers. It is endemic to open woods with moist acidic to neutral soils, and its native range is South East Canada to North Central and East USA, from Ontario to Georgia. The plants are often used as ornamental plants and folk herbal medicine. In the present study, we assembled the complete chloroplast genome sequence of *G. trifoliata* based on the next-generation sequencing method (Dong et al. 2017; Li et al. 2018). Our aims were to establish and characterize the organization of the complete chloroplast genome of *G. trifoliata* and to calibrate the phylogenetic position of *G. trifoliata* based on phylogenomic analysis.

Specimen of *G. trifoliata* was collected from Royal Botanic Gardens, Kew, U.K. (51°28′43.2″N, 0°17′44.4″W). DNA sample and voucher specimen were deposited in the Herbarium, Institute of Botany (PE), CAS with accession number BOP040568. Total genomic DNA was extracted from the dried leaves using mCTAB method (Li et al. 2013). DNA was sheared to construct 400 bp (insert size) paired-end library in accordance with the Illumina HiSeq Xten platform. The paired-end reads were qualitatively assessed and assembled with SPAdes 3.13.1 (Bankevich et al. 2012). The annotation was performed with Plann (Huang and Cronk 2015). The annotated genomic sequence had been submitted to GenBank with the accession number MK905738.

The circular chloroplast genome of *G. trifoliata* was 159,496 in length, including two short inverted repeat (IRa and IRb) regions of 26,394 bp, separated by a large single copy (LSC) region of 87,647 bp and a small single copy (SSC) region of 19,061 bp. The GC content of the whole chloroplast genome was 36.5%. The genome consisted of 112 different coding genes, of which 78 were protein-coding genes, 30 were distinct tRNA genes, and four were rRNA genes. Among these genes, a single intron was detected in 15 genes, while two genes (*ycf3* and *clpP*) were found exhibit two introns each.

A phylogenetic tree was constructed to validate the position of *G. trifoliata*. 26 species within the family Rosaceae and *Morus australis* (Moraceae) as outgroup were chosen to conduct a maximum-likelihood (ML) analysis using IQ-TREE (Nguyen et al. 2015) (Figure 1). The phylogenetic analysis revealed that *G. trifoliata* lies within subfamily Amygdaloideae and forms the progenitor lineage of the pome-bearing Maleae. The complete chloroplast genome of *G. trifoliate* fills in the vacancy of Rosaceae phylogenetic research, showing great impact on phylogenomics and comparative genomics of Rosaceae.

## Figures and Tables

**Figure 1.**
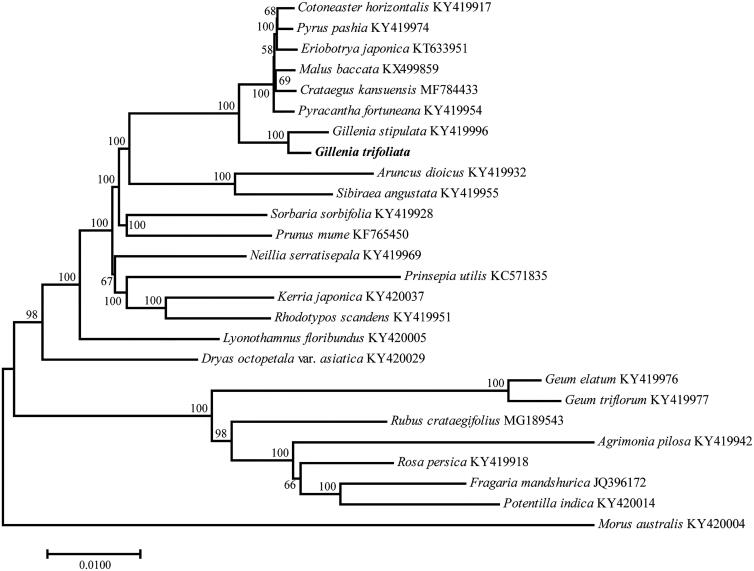
Phylogenetic tree reconstruction of 26 taxa using maximum likelihood based on complete chloroplast genome sequences. Bootstrap support values are given at each node.
